# Characterization of the truncated hemoglobin THB1 from protein extracts of *Chlamydomonas reinhardtii*

**DOI:** 10.12688/f1000research.5873.1

**Published:** 2014-12-04

**Authors:** Eric A. Johnson, Juliette T.J. Lecomte

**Affiliations:** 1 Department of Biophysics, Johns Hopkins University, Baltimore, MD, 21218, USA

**Keywords:** Hemoglobins, oxygen binding, THB1, Chlamydomonas reinhardtii

## Abstract

Truncated hemoglobins (TrHbs) belong to the hemoglobin superfamily, but unlike their distant vertebrate relatives, little is known about their principal physiologic functions.  Several TrHbs have been studied *in vitro* using engineered recombinant peptides.  These efforts have resulted in a wealth of knowledge about the chemical properties of TrHbs and have generated interesting functional leads. However, questions persist as to how closely these engineered proteins mimic their counterparts within the native cell. In this report, we examined THB1, one of several TrHbs from the model organism *Chlamydomonas reinhardtii.* The recombinant THB1 (rTHB1) has favorable solubility and stability properties and is an excellent candidate for *in vitro *characterization. Linking rTHB1 to the *in vivo* protein is a critical step in understanding the physiologic function of this protein. Using a simplified three-step purification protocol, 3.5-L batches of algal culture were processed to isolate 50–60 μL fractions enriched in THB1. These fractions of *C. reinhardtii* proteins were then subjected to physical examination. Using gel mobility, optical absorbance and immunoreactivity, THB1 was identified in these enriched fractions and its presence correlated with that of a heme molecule. Mass spectrometry confirmed this cofactor to be a type *b* heme and revealed that the native protein contains a co-translational modification consistent with amino-terminal acetylation following initial methionine cleavage.

## Introduction

Extraordinary advances in structural biology and biothermodynamics are credited to vertebrate hemoglobins (Hb). This exemplar tetrameric assembly is a component of virtually all biochemistry textbooks, which emphasize its role in reversible molecular oxygen binding. Yet, the superfamily of Hbs is distributed in all domains of life, and phylogenetic analyses have traced its origins to the Archean eon, an era that preceded by billions of years the evolution of highly specialized proteins for the storage and delivery of dioxygen. In contrast to our detailed knowledge of Hbs from animals, little information is available about other Hbs; in fact, it can be said that the cellular functions of the majority of Hbs remain open to investigation.

Sequence data organize the Hb superfamily in three ancient lineages: M (myoglobin-like, containing the vertebrate Hbs), S (sensor-like), and T (truncated) ^1^. The T lineage of interest to this report has representatives (TrHbs) in prokaryotes, fungi, and Viridiplantae ^1– 3^. Although many TrHb genes have been identified through genome sequencing, only a few have undergone biophysical examination at the protein level. Even fewer have been investigated within their native cell, in large part because of the difficulty in characterizing non-essential proteins present at low (nM) levels in organisms that cannot be cultured or are non-transformable. This “glaring lack of reliable information on function” ^4^ contrasts with the ever-growing collection of sequences and prevents a complete understanding of the determinants of reactivity in Hbs. A considerable challenge in Hb research is deciphering how evolution has harnessed the helical folds of the M and S lineages (“3/3” helical arrangement) and the T lineage (“2/2” helical arrangement) to adapt to various environments and metabolic needs.

Much insight regarding TrHbs and their relationship to other Hbs has been based on experimentation using engineered, recombinant proteins. Although indispensable for an understanding of Hb chemistry, the use of recombinant material carries an important caveat. Unless the protein is extracted from the native cell, doubts should remain regarding the identity of the cofactor, if any, that is associated with the polypeptide *in vivo*. This is especially true for the proteins of photosynthetic organisms because a variety of hydrophobic chromophores may be available ^5^ to occupy the heme cavity and perform functions independent of dioxygen binding. Co- and post-translational modifications of the polypeptide chain and the cofactor are also possible and can be assessed only with the native material. Thus, it is essential to inspect the properties of the native protein to validate the biophysical data.

THB1 is a TrHb from *Chlamydomonas reinhardtii*. This unicellular, diflagellate alga is a key model organism, used in diverse fields such as microbiology ^6^, developmental biology ^7^, photosynthesis ^8^, optogenetics ^9^, synthetic biology ^10^ and biofuels ^11^. In prior work, we prepared recombinant apoprotein based on the *THB1* gene from *C. reinhardtii*. The holoprotein was then reconstituted with a ferric *b* heme and purified *in vitro*. This recombinant THB1 (rTHB1) remains a monomer even in concentrated (mM) solutions. Using mutagenesis, optical absorbance, and nuclear magnetic resonance spectroscopy, we identified the axial ligands to the heme iron as the proximal histidine and a distal lysine in both the ferric and ferrous states. We also determined that the distal lysine can be displaced by the diatomic molecules that are common ligands to other Hbs (O _2_, CO, NO ^•^). *In vitro*, rTHB1 was found to possess the nitric oxide dioxygenase activity also exhibited by many Hbs ^12^. Interestingly, *in vivo* studies performed in parallel with these recombinant studies linked the expression of THB1 to the NIT2 transcription factor ^13^. This transcription factor is the only known positive acting regulatory factor for the induction of genes required for the nitrate assimilation pathway ^14^. The inducible control of THB1 by NIT2 strongly suggests THB1 is also involved in nitrate metabolism.

rTHB1 displays novel physico-chemical properties and is suitable for further analysis. The THB1 protein expressed *in vivo* has unique physiology and is present in an extensively characterized model organism. With these combined attributes, THB1 is an excellent protein to gain a deeper understanding of TrHbs. We describe here a traditional biochemical approach to the characterization of the protein expressed in its native environment with the goal to correlate the recombinant protein used in biophysical studies with the native protein.

## Materials and methods

All chemicals used were scientific-grade and were purchased from Sigma-Aldrich unless otherwise noted.

### Cell culture

Strain CC-1690 was obtained through the Chlamydomonas Resource Center (University of Minnesota). Cells were maintained on Tris acetate phosphate (TAP) medium agar plates ^15^ until use. Liquid cell cultures were grown in Sager-Granick M medium ^16, 17^, at 20°C under constant agitation, aeration with sterile air and illuminated with cool white fluorescent light on a 14/10 (on/off) cycle to synchronize cell growth.

### Protein isolation from *C. reinhardtii* cultures

Strain CC-1690 ( *NIT1*+, *NIT2*+, mt+) was grown in Sager-Granick M medium as described above until the culture reached a cell density of approximately 1–2 × 10 ^6^ cells/mL in a volume of 3.5 L. Algal cells were harvested by centrifugation at 5,000 × *g* for 10 min at 4°C, then washed once with 20 mM Tris–HCl pH 8.0, 20 mM NaCl and again concentrated by centrifugation at 5,000 × *g* for 10 min at 4°C. The resulting cell pellet was immersed in liquid nitrogen and allowed to equilibrate for 5 min before transfer to ice for 5 min. The frozen pellet was thawed and resuspended in 20 mL of 20 mM Tris-HCl pH 8.0 at 4°C. After resuspension, the solution was again immersed in liquid nitrogen for 5 min and transferred to ice for 5 min. The solution was thawed to 4°C and immediately separated by centrifugation at 5,000 × *g* for 15 min. The supernatant was removed and separated by centrifugation at 20,000 × *g* for 30 min at 4°C, then at 30,000 × *g* for 30 min at 4°C. The supernatant, free of any visible cell debris, was flash-frozen in liquid nitrogen and stored at -80°C until further purified.

The 20 mL of protein suspension was diluted to 50 mL using 20 mM Tris-HCl pH 8.0, 20 mM NaCl, then passed over a 2 mL HiTrap Q Fast Flow column by fast protein liquid chromatography, FPLC, (GE Healthcare Life Sciences) using a flow rate of 1 mL/min. Following a brief wash with 20 mM Tris-HCl pH 8.0, 20 mM NaCl, protein was eluted from the column using a 20 mM–400 mM NaCl gradient run over the course of 1 h. Protein was collected from this gradient in 3-mL fractions and each fraction tested for the presence of THB1 by dot-blot immunodetection using custom-made rabbit polyclonal antibodies (Covance) raised against THB1 ^13^. These antibodies were used at 1:10,000 dilution in TBS buffer with 5% milk. THB1 was found to elute from the column at approximately 100 mM NaCl. Positive fractions were pooled and concentrated to 2.5 mL using a 4-mL centrifugal concentrator with a 10,000 Da molecular weight cut-off. The resulting protein solution was separated on a 16/600 Superdex 75 column (GE Healthcare Life Sciences) by FPLC using a 1 mL/min flow rate. 1-mL fractions were collected and tested for THB1 using immunodetection as above. THB1 was found to elute from the column approximately 35 mL after the 40 mL void volume of the column. Positive fractions were pooled and again concentrated using a 4-mL centrifugal concentrator with a 10,000 Da molecular weight cut-off. The sample was concentrated to the stop volume of the concentrator, approximately 50–60 μL. The protein extract was stored at 4°C and used within 48 h.

### Gel electrophoresis, protein transfer and detection

Protein samples were analyzed by native or denatured gel electrophoresis. Native electrophoresis used precast Any-kD TGX polyacrylamide gels (Bio-Rad) and 25 mM Tris, 192 mM glycine pH 8.3 in both the upper and lower reservoirs. The proteins were separated at 4°C and 100 V constant voltage for 2 h. Following the completion of the electrophoresis, the proteins were transferred to 0.45 μm nitrocellulose (Whatman) using a wet-tank transfer with 25 mM Tris, 192 mM glycine and 10% methanol as the transfer buffer. The transfer was performed at 4°C and constant amperage of 300 mA for 60 min. Following transfer, the nitrocellulose blot was washed once with distilled water. The presence of heme was detected by coating the surface of the nitrocellulose with a thin layer of ECL reagent (Immobilon Western Chemiluminescent HRP Substrate, Millipore) followed by imaging on a chemiluminescent imager (ProteinSimple). The nitrocellulose was then washed with water and the bound protein was detected using MemCode protein stain (ThermoScientific) as per manufacturer’s instructions. The blot was again imaged. The nitrocellulose was destained as per manufacturer’s instructions and blocked using TBS buffer with 5% milk for 30 min. The blot was then probed with polyclonal rabbit anti-THB1 antibodies at 1:10,000 dilution as described above and previously ^13^.

Denaturing gel electrophoresis was performed using precast 16.5% Tris-tricine (Bio-Rad) with 100 mM Tris, 100 mM Tricine and 0.1% SDS in both the upper and lower reservoirs. The voltage was a constant 100 V for 1.4 h. Proteins were then either stained with Silver Stain Plus (Bio-Rad) according to manufacturer’s instructions or transferred to nitrocellulose and used for immunostaining as described above with polyclonal antibodies against THB1 ^13^, histone H3 (AbCam, Cambridge Massachusetts ab1791), nitrate reductase (Agrisera, Sweden AS08310) or β–subunit of the ATP synthase (Agrisera, Sweden AS03030).

### Mass spectrometry

Following purification and concentration, a 10-μl sample was injected into a Waters Acquity/Xevo-G2 UPLC-MS and analyzed using reverse-phase chromatography on a Waters BEH C4 (2.1 mm × 50) column (300 Å, 1.7 µm resin) using a mix of water/acetonitrile (in 0.1% formic acid) over a 10 min separation period. The solvent profile was 0% acetonitrile, ACN, (0–1 min), gradient to 80% ACN (1–7.5 min), hold at 80% ACN (7.5–8.5 min) and 0% ACN (8.5–10 min). Following chromatography, the sample was immediately injected into the QTof MS/MS mass spectrometer. A locked mass was used to calibrate found masses within the sample using the intermittent injection of a known sample of leu-enkephalin (M _r_ 556.2771) continuously throughout the analysis. The data collected during the analysis were processed using the BioPharmaLynx software package (Waters).

### Optical spectroscopy

Following the purification and concentration described above, a 2 μL sample was placed onto a NanoDrop 2000c spectrometer (ThermoScientific) and spectra obtained according to manufacturer’s instructions.

## Results

The unicellular green alga *C. reinhardtii* contains approximately 140 mg of total protein per 1 g cells ^15^. Previous work has identified THB1 within these cells, but both immunodetection results and qPCR of gene transcripts suggest that THB1 is not an abundant cellular component. We determined that the *THB1* gene is regulated by the *NIT2* gene for nitrogen metabolism ^13^, and therefore for these experiments selected the wild-type strain of *C. reinhardtii* (CC-1690 also called 21 gr) known to contain the *NIT2* gene (unlike the common laboratory strains CC-124 or CC-125 also called 137c). Owing to this low induced protein expression, isolation of the protein from whole cells of *C. reinhardtii* requires dramatic enrichment of THB1 and exclusion of the vast majority of the cell’s other proteins. The approach taken in this report comprises three steps that avoid the use of detergent and enrich progressively a fraction of the total cell protein with THB1. Although total purity was not achieved by the method, enough surrounding protein could be removed so that physical aspects of THB1 were identified within the pool of remaining proteins.

**Figure 1.**
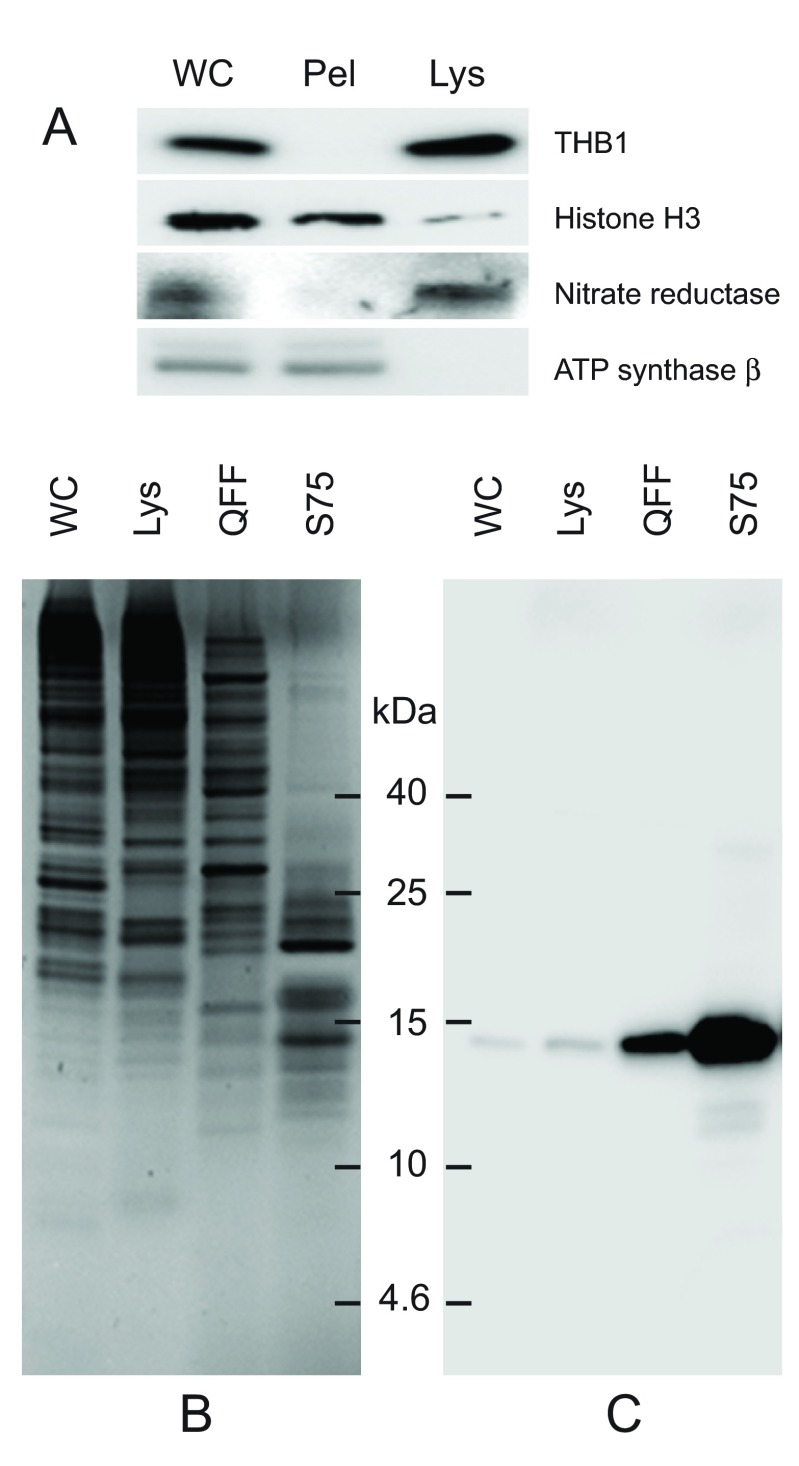
Purification of THB1 from *C. reinhardtii* cell culture. Protein samples from different stages of purification were analyzed by electrophoresis. ( **A**) Samples of protein extracts before and after lysis of *C. reinhardtii* cells with liquid nitrogen. Proteins separated on 16.5% Tris-tricine gel then transferred to nitrocellulose and immunostained with antibodies against proteins localized to different cellular compartments. Lysis by liquid nitrogen enriches the lysate with soluble proteins without enrichment of proteins found in major algal organelles. Histone H3 protein is located in the nucleus, nitrate reductase is a soluble cytosolic protein and the ATP synthase β subunit is part of the thylakoid membrane of the chloroplast. ( **B**) Samples from different steps in the purification procedure were separated by electrophoresis. Following separation, the proteins within the gel were visualized using silver stain. ( **C**) Proteins prepared identically to those detected in panel B were transferred to nitrocellulose followed by immunostaining with polyclonal antibodies specific for THB1. In addition to the protein samples, a lane was used for molecular weight markers (Spectra LR, ThermoScientific). The numbers indicated between the panels represents location of the markers (kDa molecular mass). WC, whole cell protein extract. Lys, protein extract lysate, and Pel, protein pellet following liquid nitrogen fracturing. QFF, concentrated sample following anion exchange chromatography. S75, concentrated sample following separation on the Superdex 75 column.

The first enrichment step involves the freeze fracturing of whole cells. Following low-speed sedimentation, cell pellets are flash-frozen in liquid nitrogen that effectively ruptures the cell without severely disrupting its membranes. Following rupture, soluble proteins partition to the lysate, while proteins bound to membranes or within larger organelles are removed through a series of centrifugation steps ( Figure 1A). This facilitates the release of the soluble proteins while retaining, and disposing of, the majority of the contents within the cell body. This gentle procedure also leaves pigmented complexes predominantly associated with larger membrane structures that do not partition to the lysate. As THB1 does partition to the lysate, this result suggests that in the cell, THB1 is a soluble protein similar to its recombinant counterpart (see introduction). Following the isolation of the lysate, the soluble cellular proteins are further enriched by anion-exchange chromatography ( Figure 1B, lane 3). Previous work with rTHB1 has shown that the protein tends to bind weakly to anion-exchange resins ^13^. When the soluble cellular proteins were passed over the resin, THB1 did bind but eluted early when a salt gradient was applied to the column. Further enrichment involved size-exclusion chromatography, which both enriched THB1 ( Figure 1C, lane 4) and excluded other proteins with molecular weights substantially larger than that of THB1 ( Figure 1B, lane 4).

**Figure 2.**
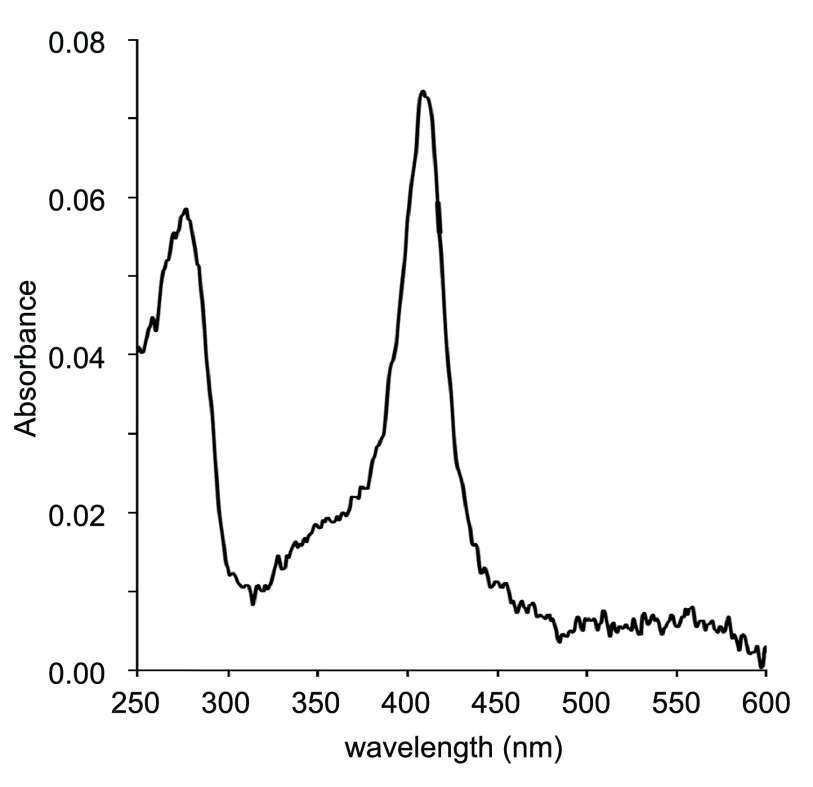
Optical spectroscopy following purification. A 2-μL drop of the concentrated fraction following separation on Superdex 75 was scanned using a nanodrop spectrometer (average of four scans). Maximum absorbance occurs at 410 nm and corresponds to the Soret peak of rTHB1 at neutral pH in the ferric state ^13^. Based upon the published extinction coefficient for the recombinant protein under these conditions, this sample contains approximately 5 μM THB1. Note that the presence of ferric rather than ferrous protein may be the result of oxidation during the purification procedure. Q bands (500–600 nm) are not detected because of the low signal-to-noise ratio.

The protein fraction following Superdex 75 reduced an initial volume of 3.5 L of cells to yield approximately 50–60 μL of isolated proteins. The protein solution was visibly light pink in color, and its optical spectrum ( Figure 2) showed a distinct absorption maximum corresponding to the known Soret band for ferric THB1 (410 nm). Using the estimated extinction coefficient for this form of THB1 ^13^, it can be estimated that the solution was approximately 5 μM in THB1.

**Figure 3.**
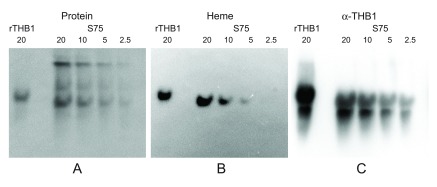
Native gel electrophoresis of purified proteins and detection of heme. Following separation on the Superdex 75 column and concentration, the sample (identical to the sample used in Figure 2) was separated on an acrylamide gel under native conditions and transferred to nitrocellulose. The same nitrocellulose was stained to detect ( **A**) total protein, ( **B**) heme and ( **C**) THB1 as described in the methods section. Dilutions of the sample were used owing to differing sensitivities of the different detection methods with immunodetection being much more sensitive than the heme or protein stain. The immunodetection using anti-THB1 antibodies therefore has high background even at very short exposure times; however, only a single protein band co-localizes with both heme stain and THB1 supporting that THB1 is associated with heme. Quantities are marked in pmol above each lane.

The absorbance spectrum of the solution matched that of a heme protein. However, a direct link of this pigment and THB1 is not established since the solution still contains other proteins in addition to THB1. To investigate the colocalization of THB1 with any bound cofactor, native electrophoresis can be used, as tightly bound cofactors will remain associated with the protein while it migrates through the gel. Following Superdex 75 chromatography the proteins in the resulting solution were separated using native gel conditions and then transferred to nitrocellulose. Protein staining ( Figure 3A) shows that the various components of the samples were separated within the native gel. Chemiluminescence “heme stain” ^18, 19^ ( Figure 3B) displays the presence of a single band. Following protein staining and heme staining, the same nitrocellulose blot can again be used for immunostaining with polyclonal antibodies against THB1 ( Figure 3C). Immunostaining is much more sensitive than heme staining, and very short exposure times were sufficient to detect stained bands on the nitrocellulose. The procedure revealed a major band at the same position as the heme signal was obtained. In addition, rTHB1 (with known heme content) reacts with both the heme stain and the immunostain, although when electrophoresis was performed under native conditions, the recombinant THB1 had a slightly lower mobility in the gel than the native protein.

**Figure 4.**
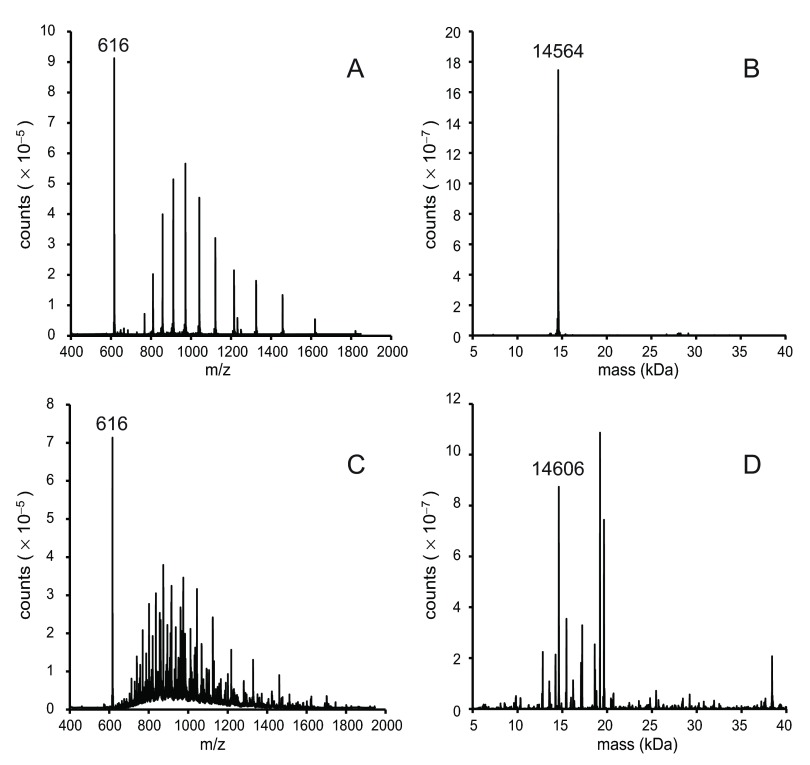
Mass spectrometry of purified proteins. A 10-μL sample was separated on a C4 column and detected as intact protein by mass spectrometry as described in the methods section. ( **A**) The unprocessed mass spectrum of rTHB1 as represented by the relative signal intensity at each mass-to-charge value between 400 and 2000. ( **B**) The same data used to generate the spectrum in panel **A** were subject to deconvolution using the BioPharmaLynx software program to determine the mass of intact proteins within the sample. ( **C**) The unprocessed mass spectrum (as in panel **A**) of the concentrated sample from *C. reinhardtii* following separation on the Superdex 75 column. ( **D**) The data shown in panel **C** were subjected to processing as in panel **B**. panels **B** and **D** were processed under the same conditions.

The components of the Superdex 75 fraction were investigated using mass spectrometry. To establish a protocol, the procedure was first performed with the recombinant protein. rTHB1 elutes from the C4 column at approximately 4.9 min using the conditions described in the methods section. The spectrum of the recombinant protein, displayed as the ratio of relative mass to charge number ( *m*/ *z*) ( Figure 4A), shows an *m*/ *z* distribution for a protein and a single strong *m*/ *z* peak at 616. This single *m*/ *z* signal is consistent with that of the *b* heme (molecular mass 616 Da) bound to the recombinant THB1. The relative mass of the protein (apo rTHB1) is 14564 ( Figure 4B), as expected if the initial methionine is cleaved.

When the mass analysis was repeated on the purified proteins from *C. reinhardtii* the *m*/ *z* spectrum was complicated by the presence of additional proteins ( Figure 4C). One notable similarity between the two samples is the strong, single signal at *m*/ *z* = 616. This suggests that a molecule that exhibits the same *m*/ *z* as the *b* heme in Figure 4A is among the purified algal proteins, further supporting the hypothesis that this sample contains *b* heme bound within the native THB1. Deconvolution of the purified protein spectrum ( Figure 4D) reveals a group of molecules whose distribution of molecular mass roughly correlates to the distribution of protein bands seen in the silver-stained denatured protein electrophoretic gel ( Figure 1B). Examination of these individual relative masses failed to find any with a value of 14564 corresponding to the recombinant THB1. There was, however, a significant signal at a mass of 14606, which represents an increase of 42 above the rTHB1 relative mass. We concluded that the 14606 signal did correspond to THB1, modified by the acetylation of a single functional group, which replaces a hydrogen atom by a COCH _3_ moiety. We attribute the slightly different migrations of rTHB1 and THB1 in the gel of Figure 3C to this modification of the native protein.

In previous work, THB1 was found within the soluble protein extracts of *C. reinhardtii* flagella ^20^. The protein was identified by excision from polyacrylamide gels, followed by protease digestion and peptide mass spectrometry. Two peptides from this extracted protein were attributed to THB1, one of them encompassing the 13 N-terminal residues of the protein. Re-examination of the peptide mass spectrometry data showed the assignment of N-terminal acetylation to this fragment (George Witman, personal communication). In light of this additional information, it seems most likely that the increased molecular weight seen in native form of THB1 comes from acetylation of the N-terminal peptide, in this case an alanine (Ala2). In sum, the mass spectral data obtained on the intact protein indicate that THB1 is present in the *C. reinhardtii* protein extract, that it lacks its initial methionine, has N-terminal acetylation, and that a *b* heme is associated with the protein. 

*In vitro* experiments have shown that TrHbs (and many Hbs in general) are capable of processing reactive nitrogen species to less toxic molecules. This activity has been specifically shown in rTHB1 ^13^, which converts nitric oxide to nitrate via the nitric oxide dioxygenase (NOD) reaction ^12^. As an additional assessment of the properties of the native protein, we attempted to assay this activity on material generated after passage through the Superdex 75 column. The small sample volumes complicated measurement of NOD activity and necessitated the use of microliter spectrophotometry. Given these limitations, it was not possible to determine accurate yields of nitric oxide conversion. The results of these attempts, although not presented because of high background and low number of replicates, did not conflict with the high yields observed with rTHB1 ^13^, and therefore NOD activity is still a plausible role for THB1 *in vivo*.

Mass spectrometry data of purified proteins Mass spectrometry data performed to analyse the components of the Superdex 75 fraction are reported. The procedure was first performed with rTHB1.

## Discussion

To date hundreds of TrHb genes have been identified within the genomes of bacteria, fungi, plants and algae ^1, 2^. Despite the accumulation of sequences and remarkable progress in genetic and structural knowledge, there is limited information about the physiological role of these proteins within most organisms. In some photosynthetic prokaryotes, there is evidence that constitutive expression of TrHbs helps the cell survive exposure to reactive oxygen species (ROS) ^21^ or reactive nitrogen species (RNS) ^22^. The connection between TrHbs and ROS/RNS mediation has also been identified within non-photosynthetic organisms such as *Mycobacterium tuberculosis*, an organism in which TrHbs are linked to protection of the cell from the cytotoxic effects of external NO ^•^^23, 24^. The cytotoxic effects of internal NO ^•^ maybe mitigated in some plants and algae where TrHbs are induced under hypoxic stress ^25– 27^. The study of TrHbs therefore helps our understanding of both intracellular stress and intercellular “redox warfare” ^28^. Ideally TrHbs may be targets for the design of synthetic molecules manufactured to control these events, for therapeutic benefit in humans or economic benefit in plants and algae.

THB1 from *C. reinhardtii* provides a welcome opportunity to expand the examination of the TrHb lineage – THB1 is present in a very well characterized organism, the native protein is linked to a critical metabolic pathway involving RNS, and the recombinant protein is amenable to detailed *in vitro* studies. These *in vitro* studies are essential because properties that dictate chemistry, such as the identity of the distal ligand to the heme iron, the ability to bind exogenous molecules, and the iron redox potential, cannot be inferred from the amino acid sequence and are best determined using large quantities of pure material with and without isotopic labels or amino acid replacements. However, in order to extrapolate *in vitro* findings to *in vivo* conditions, it is imperative to question how well the recombinant wild-type model mimics the native protein. In the experiments presented here, we have sought to bridge the *in vitro* and *in vivo* studies by comparing the physical characteristics of rTHB1 to those of the protein from the native cell.

Our study revealed one difference between the THB1 polypeptides synthesized by *E. coli* and *C. reinhardtii*. In many eukaryotic cells the majority of cytoplasmic proteins are N-terminally acetylated ^29^ for purposes that are not entirely understood ^30^. N-terminal (Nt) acetylation has also been observed in *C. reinhardtii*, though studies have focused on chloroplast targeted proteins ^31^ with the conclusion that the modification may retard degradation. We have found that THB1 is acetylated, most likely at the N-terminus. Whether this modification contributes to the stability of cytosolic proteins such as THB1 is not known. The first two eukaryotic TrHbs to be isolated from their native organisms (the ciliates *Paramecium caudatum* and *Tetrahymena pyriformis*) exhibit a blocked N-terminus when subjected to amino acid analysis. The blocking group was identified as an acetyl moiety in *P. caudatum* globin ^32^ and inferred in *T. pyriformis* globin ^33^. Thus, of the three native eukaryotic TrHbs peptides so far characterized, all three appear to contain the same modification, though its consequences remain to be determined. 

The three-dimensional structure of several relatives of THB1 has been solved. These include globin domains from *P. caudatum* (PDB ID 1DLW), two cyanobacterial species ( *Synechocystis* sp. PCC 6803, PDB ID 1RTX and *Synechococcus* sp. PCC 7002, PDB ID 4MAX) and *Chlamydomonas eugametos* (PDB ID 1DLY). Nuclear magnetic resonance (NMR) data collected on rTHB1 ^13^ indicate that the heme domain is structurally similar to other characterized TrHbs. Structural data (NMR and X-ray crystallography, Matthew Preimesberger and Selena Rice, personal communication) also concur that the N-terminal extension is disordered (data not shown). This leads us to assume that acetylation has minimal impact on the reactivity and enzymatic properties of the native protein relative to its recombinant counterpart.

Similarity between recombinant and native THB1 proteins extends to the holoprotein forms. The nature of bound cofactors is a critical feature of any enzyme. For hemoglobins, the “standard” cofactor is a *b* heme (Fe-protoporphyrin IX), but instances of covalent heme attachment have been reported in *Synechocystis* ^34^ and *Synechococcus* ^22^ TrHbs. This or other types of post-translational modification may occur in TrHbs and should not be overlooked. More importantly, many derivatives of the tetrapyrrole core and other large hydrophobic molecules are present in living cells, especially photosynthetic ones. The 3/3 globin fold is known to accommodate several such molecules, for example chlorophyllin ^35^, chlorophyllide ^36^, and open tetrapyrroles ^37– 39^, and the same might be expected of the 2/2 proteins. Our results establish that native THB1, as extracted from *C. reinhardtii* cells by gentle methods, contains an unmodified *b* heme as was incorporated into the recombinant apoprotein to generate rTHB1. In addition, no significant quantity of a species with mass indicating covalent attachment of the heme was detected. It must also be noted that although we isolated holo THB1, we cannot rule out the possibility that some amount of (functional) apoprotein is present or can exist under different growth conditions. Further examination of the protein within the cell would be required to address this question.

The results presented here compared the recombinant and native versions of the THB1 protein from *C. reinhardtii*. Both proteins exhibit similar optical properties; both are recognized by the same antibody, exhibit similar migration upon gel electrophoresis and possess the same cofactor. Co-translational modification (acetylation) occurs to the native protein, but the location of the modification in the three-dimensional structure suggests the modification’s role (if any) is regulatory and does not impact the protein’s activity. Taken together, the data reinforce the use of rTHB1 as a model for the native protein within *C. reinhardtii*. Future work will continue to utilize *in vitro* and *in vivo* experiments with the goal of understanding the activity of TrHbs from both the molecular and physiologic perspectives.

## Data availability

*F1000Research*: Dataset 1. Mass spectrometry data of purified proteins, 10.5256/f1000research.5873.d39884 ^40^
