# Supplementary material for: Characterization of the truncated hemoglobin THB1 from protein extracts of Chlamydomonas reinhardtii
Source: F1000Res. 2014 Dec 4;3:294. [Version 1] doi: 10.12688/f1000research.5873.1 (PMC4304232; doi:10.12688/f1000research.5873.1)
Supplement: Peer review history

# Peer review: Referee response for version 1

**DOI:** 10.5256/f1000research.6275.r6904

**Published:** 2015-01-12

**Authors:** Suman Kundu

**Affiliations:** 1 Department of Biochemistry, University of Delhi, New Delhi, India

Expanding genome sequence data has led to the discovery of a plethora of hemoglobins from diverse organisms through bioinformatics analysis of signature sequences. The verification of putative hemoglobins from such genomes, however, came only from investigation of recombinant hemoglobins in heterologous system. For way too long we have dabbled in understanding of biochemical, kinetic and structural properties of such recombinant hemoglobins. While such investigations have unraveled key insight and unique features of the novel hemoglobins with hypothesis for putative functions *in vivo*, a final word on their function is still awaited. The non-essentiality of these genes, their low physiological concentrations and lack of obvious relations to phenotypes has also kept an insight into their functional roles at bay from occasional *in vivo* genetic studies. A potential source of further insight into these proteins might thus lie in their post-translational modifications, modifications of the heme or their ability to interact with other key proteins *in vivo*, thus playing key roles at the proteome level. The investigation by Johnson and Lecomte addresses this key issue by characterization of an algal hemoglobin from native source in comparison to its recombinant counterpart. The experiments have been beautifully executed and the conclusions carefully laid down. While several properties of the native protein are indeed similar to the recombinant version, the N-terminal acetylation of the native hemoglobin indeed reveal new opportunities in understanding the function of these classes of proteins. It would have been exciting to see similarities or differences in the kinetic properties (on rates, off rates and ligand binding affinities) of the recombinant and native algal hemoglobins, which can provide additional information into their ligand-dependent functional roles. I am sure that the authors themselves or some other group will investigate this aspect as well in the near future. I am hopeful that this pioneering investigation will inspire other similar studies taking us into yet unknown territories of hemoglobin features and functions.

I have read this submission. I believe that I have an appropriate level of expertise to confirm that it is of an acceptable scientific standard.

# Peer review: Referee response for version 1

**DOI:** 10.5256/f1000research.6275.r6902

**Published:** 2014-12-31

**Authors:** Thomas Gorr

**Affiliations:** 1 Institute of Veterinary Physiology Vetsuisse-Faculty, University of Zurich, Zürich, Switzerland

Unraveling the functional properties of truncated hemoglobins (TrHBs) is both important and revealing as these mini globins of todays cyanobacteria and green algae not only function on the basis of a simplified two-on-two helical topology (in contrast to the three-on-three helices of canonical globins) but also link back to primordial globins that first appeared some 3 billion years ago in a biosphere virtually free of oxygen. Perhaps in reminiscence of this ancestry, many TrHBs function indeed in roles related to reactive nitrogen usage rather than as dioxygen sensing and/or transporting heme-binding proteins. A previous publication by the same authors (i.e. Johnson *et al., *2014) documented efficient NO ^•^ dioxygenase activity along with proximal histidine and displaceable distal lysine heme ligands for the recombinant THB1 (rTHB1) truncated hemoglobin of the unicellular algae *Chlamydomonas reinhardtii*. However, to further our functional understanding of TrHBs it is important to elicit how closely recombinant proteins mimic their counterparts within the native cell. Direct investigations of native TrHBs are rendered difficult due to low level (nM range) expression of these proteins and the fact that many of these organisms can neither be cultured nor transformed. To address this issue, the current publication by Drs. Johnson and Lecomte aims to link biochemical properties of *Clamydomonas* rTHB1 to the *in vivo *protein.

By using a straightforward 3-step purification protocol including freeze-thaw separation of soluble proteins followed by anion-exchange and Superdex size-exclusion FPLC chromatography steps, the authors were able to enrich native THB1 to micromolar concentrations, as judged by light absorption of the 410nm Sorret band for ferric THB1. Native gel electrophoresis and chemiluminescent ECL staining of the final Superdex purification fraction confirmed presence of a single heme-bound protein band that was positively identified as THB1 by subsequent immunostaining with self-generated polyclonal anti-THB1 antibodies (Johnson et al. Biochemistry 2014, 53, 4573-4589). Recombinant THB1 used as electrophoretical reference revealed a slightly lower mobility than the native globin. Presence of a 616 Da *b* heme (Fe-protoporphyrin IX), lack of the initial methionine and N-terminal acetylation at Ala2 was all demonstrated for the native protein (Mw=14606 Da) by UPLC-MS, again in comparison with rTHB1. As expected, the recombinant protein (Mw=14564 Da; ΔMw (native-recombinant)=42 Da; corresponds to mass increase by COCH _3_ moiety in replacement of one hydrogen atom) was devoid of any post-translational modification (acetylation). This missing acetyl moiety in rTHB1 might also explain the slightly different electrophoretic mobility in native gels.

An N-terminal amino acid sequence, blocked for Edman degradation by an acetyl moiety, has also been detected or inferred for TrHBs of the clitated protozoans *Paramecium caudatum* and *Tetrahymena pyroformis*.  Thus, of the three native eukaryotic TrHbs peptides so far characterized, all three appear to contain the same modification, though its consequences remain to be determined. Based on unpublished NMR and X-ray crystallography results by Drs. Preimesberger and Rice (pers. communication), the N-terminal extension, however, appears to be disordered and of minimal impact on reactivity and enzymatic properties in TrHbs.

Together, this publications contains an easy-to-implement protocol for the purification of low level expressed native TrHbs and, as such, represents an important contribution for further links of the biochemical properties of recombinant and native truncated hemoglobins.

I have read this submission. I believe that I have an appropriate level of expertise to confirm that it is of an acceptable scientific standard.

# Peer review: Referee response for version 1

**DOI:** 10.5256/f1000research.6275.r6903

**Published:** 2014-12-16

**Authors:** Cinzia Verde

**Affiliations:** 1 Institute of Protein Biochemistry, National Research Council, Naples, Italy

In their Research article "Characterization of the truncated hemoglobin THB1 from protein

extracts of *Chlamydomonas reinhardtii*", Johnson & Lecomte describe the structural properties of a truncated globin, purified by traditional biochemical approaches, from the extracts of *Chlamydomonas reinhardtii* with the aim to correlate the recombinant protein previously studied by biophysical techniques with the protein expressed in its native environment.

The report is comprehensive and up to date and will certainly become a key reference in the field. The topic is of interest to many readers and to my knowledge has not been covered by many articles. This article highlights the similarities and differences between recombinant protein over-expressed in Escherichia coli and native protein. Although extensive research has been conducted to investigate the physiological function of truncated globins, knowledge of their functional role is limited by the lack of facile methods for *in vivo* studies. Consequently, the most telling insights into the physiological role of these proteins have arisen via classical approach on the recombinant protein. As the authors note, potential correlation between recombinant and native proteins may help to formulate plausible mechanisms for the function of these globins, and this is a particularly interesting highlight of this work. The paper is short but very well written, with a balanced view of the literature and makes for interesting reading. I have no comments to offer as to how to improve the paper and recommend indexing as is.

I have read this submission. I believe that I have an appropriate level of expertise to confirm that it is of an acceptable scientific standard.

# Peer review: Referee response for version 1

**DOI:** 10.5256/f1000research.6275.r6905

**Published:** 2014-12-16

**Authors:** Michael Berenbrink

**Affiliations:** 1 Institute of Integrative Biology, University of Liverpool, Liverpool, Canada

This is a solid study, addressing an important point in the study of truncated haemoglobins (and indeed all proteins), namely the verification of results obtained on recombinant proteins also with proteins obtained in the native state. The experimental design and methods are appropriate, the results are clearly presented and carefully discussed, and the conclusions are justified and supported by the data.

I have read this submission. I believe that I have an appropriate level of expertise to confirm that it is of an acceptable scientific standard.
